# Effects of osthole and *Bacillus amyloliquefaciens* on the physiological growth of *Panax quinquefolius* in a forest

**DOI:** 10.3389/fmicb.2024.1497987

**Published:** 2024-12-12

**Authors:** Jinhui Jiang, Guangxiong Fan, Rong Wen, Tao Liu, Shuran He, Shengchao Yang, Shuhui Zi

**Affiliations:** ^1^College of Agronomy and Biotechnology, Yunnan Agricultural University (YNAU), Kunming, China; ^2^College of Resources and Environment, Yunnan Agricultural University (YNAU), Kunming, China; ^3^College of Biological and Agricultural Sciences, Honghe University, Mengzi, China

**Keywords:** osthole, *Bacillus amyloliquefaciens*, *Panax quinquefolius*, microbiome, resistance

## Abstract

**Introduction:**

The biological activities of osthole have been widely reported in recent years. However, few studies have been conducted on osthole in agriculture, and its effects on plant growth have little been reported.

**Methods:**

Three experimental treatments were set up in this experiment: blank control (CK), osthole (CLS), and *B. amyloliquefaciens* (LKWS). In this study, the effects of osthole and *Bacillus amyloliquefaciens* on the growth parameters, photosynthesis, antioxidant enzyme activities, disease incidence, and microbiome of forested *P. quinquefolius* were tested.

**Results:**

This study demonstrates that the use of osthole and *B. amyloliquefaciens* significantly improved the growth of *Panax quinquefolius* in a forest compared to that in the control treatment, increased the total chlorophyll and carotenoid content of *P. quinquefolius*, significantly increased its net photosynthetic rate, and decreased the stomatal conductance and intercellular CO_2_ levels. In addition, the use of osthole and *B. amyloliquefaciens* significantly improved ascorbate peroxidase and peroxidase (POD) activities, enhanced antioxidant activities of the *P. quinquefolius* POD, and reduced the disease incidence and index of American ginseng anthracnose. Based on the American ginseng microbiome analysis, the use of osthole and *B. amyloliquefaciens* could change the structure of the American ginseng microbial community, significantly increase the diversity of American ginseng bacteria, significantly decrease the diversity of American ginseng fungi, stimulate the recruitment of more growth-promoting microorganisms to American ginseng, and build a more stable microbial network in American ginseng.

**Discussion:**

In conclusion, we found that the application of osthole had a positive effect on the growth of American ginseng, providing a theoretical basis for its subsequent application in agriculture.

## 1 Introduction

Osthole, also known as osthol (7-methoxy-8-(3-methyl-2-butenyl)-2H-1-benzopyran-2-one; C15H16O3), is a natural coumarin first derived from *Cnidium* plant. The mature fruits of *Cnidium* contain high levels of osthole, which is commonly used in the clinical practice of traditional Chinese medicine, and osthole is also widely found in other medicinal plants (Zhang et al., 2015). Previous studies have shown that osthole has many pharmacological properties, including anti-inflammatory (Liao et al., 2010) and anti-osteoporosis (Kuo et al., 2005) effects, and can inhibit systolic blood pressure (Ogawa et al., 2007), Alzheimer’s disease (Kuo et al., 2005) and cancer (Shokoohinia et al., 2018). Osthole has long been used as an antifungal agent and insecticide against plant diseases and pests in China due to it exhibiting less harmful effects on humans and the environment (Ren Z. et al., 2020; Zhang et al., 2016). In addition, *Bacillus amyloliquefaciens*, as a biofertilizer, provides significant help in increasing agricultural yields and plant resistance.

*Bacillus amyloliquefaciens* is a gram-positive spore-forming bacterium found in the soil that can colonize the inter-roots of plants and grow under stressful conditions. It has been defined as a non-toxic and environmentally friendly plant growth-promoting agent (Chen et al., 2007; Qiao et al., 2014). As a plant growth-promoting inter-root bacterium, *B. amyloliquefaciens* is considered an excellent agent for exploring biofertilizers and biocontrols in agriculture, and is used to improve plant tolerance to biotic and abiotic stresses (Dimopoulou et al., 2021; Gamez et al., 2019; Kazerooni et al., 2021).

*Panax quinquefolius* is native to the eastern temperate forest regions of North America and was first discovered in Quebec, Canada. American ginseng has been cultivated in China since the 1980s (McGraw et al., 2013). After more than 40 years of development, China has become the third largest country for *P. quinquefolius* cultivation (Huang et al., 2013). American ginseng is a well-known medicinal plant with a high market demand as dietary supplements and functional foods (Pang et al., 2023). American ginseng is known for its wide range of pharmacological effects, including anticancer, antioxidant, anti-aging, anti-fatigue, memory-enhancing, and immune-enhancing effects (Cheong et al., 2014; Hwang et al., 2014; Kim et al., 2018; Riaz et al., 2019; Tan et al., 2013). Various chemical components, including ginsenosides, lipids, polysaccharides, organic acids, amino acids, phenolic acids, and vitamins, have been identified in *P. quinquefolius* that exert various effects (Guo et al., 2015; Lin et al., 2019; Wang et al., 2015). In addition to specific advantages over field farming, such as lower costs and often higher prices, American ginseng forest plantations can improve forest health and increase biodiversity (Sheban et al., 2022).

In order to better utilize the growth-promoting effects of osthole and *Bacillus amyloliquefaciens* in the cultivation of *Panax quinquefolius*, the present study aimed to (1) investigate the effects of osthole and *Bacillus amyloliquefaciens* on the growth, photosynthesis, and antioxidant systems of *Panax quinquefolius* in the understory, and (2) to determine the regulatory effects of osthole and *Bacillus amyloliquefaciens* on the endophytic bacterial communities in *Panax quinquefolius*, and to explore the potential mechanisms of its role in the promotion of plant growth. The results of this study are expected to provide an important scientific basis for further application of osthole in agriculture and practical suggestions for optimizing the cultivation and management of American ginseng.

## 2 Materials and methods

### 2.1 Materials

Osthole was purchased from Chengdu New Sunrise Crop Science Co., Ltd. (Chengdu, China), with the main ingredient of 0.4% osthole used in subsequent tests. A *B. amyloliquefaciens* strain was purchased from Zhongnong Lukang (Beijing) Biotechnology Co. (Beijing, China).

### 2.2 Plant growth conditions

The experiment was conducted in 2022 at a forested American ginseng base in Luquan County, Kunming, Yunnan Province, China. The site is located at 25°38′ N latitude and 102°80′ E longitude, covering an area of approximately 50,000 m^2^, with an elevation of 2,700 m. The average annual temperature is approximately 7.0–10.9°C, ≥10°C effective cumulative temperature is 3,300°C, and annual precipitation is 1,000 mm. Precipitation in the months of June–August accounts for approximately 70% of the annual precipitation. The test material comprised 2-year-old understory western ginseng, with a plant spacing of 7 × 20 cm in a north–south direction.

### 2.3 Experimental design

The randomized group method was used, and three treatments set up: blank control (CK), osthole (CLS), and *B. amyloliquefaciens* (LKWS). Three replications and 2-year-old American ginseng seedlings were used, isolation boards set up between the treatments to prevent mutual influences, and the treatments initiated from June 1. Treatments were performed every 15 days for a total of five treatments and processed up until July 30; all treatment concentrations used were in accordance with instructions and product manuals of the corresponding companies. CLS (7.720 mL) was diluted 400 times, and LKWS (21.45 g) diluted 700 times.

### 2.4 Plant morphological indicators

The agronomic traits of American ginseng were measured at the leaf spreading stage. The main metrics measured were plant height, stem thickness, root thickness, and root dry weight. Plant height was measured by placing a tape measure from the bottom of the ginseng to the top of the first leaf. Stem and root thicknesses were measured at the thickest point using a vernier caliper. At harvest, the roots were uprooted and washed with distilled water, where after they were dried at 70°C for 1 week. When the weight was almost unchanged, the dry weight of the roots was weighed with an electronic balance of one ten-thousandth of a millimeter.

### 2.5 Plant physiological indicators

#### 2.5.1 Measurement of photosynthesis

Stomatal conductance (*G_s_*), intercellular CO_2_ concentration (*C_i_*), net photosynthetic rate (*P_n_*), and transpiration rate (*T_r_*) of *P. quinquefolius* were measured during its flowering period using an LI-6400 portable photosynthesis assay and photosynthesizer under sunny and cloudless weather conditions and between 10:00 and 12:00 Beijing time.

#### 2.5.2 Determination of chlorophyll content

Leaves were collected on the same day, immediately placed into liquid nitrogen, and returned to the laboratory to be stored in a − 80°C refrigerator until the chlorophyll content was measured. The chlorophyll content of *P. quinquefolius* was determined in strict accordance with the instructions of a chlorophyll kit (G0601F) supplied by Suzhou Grace Biotechnology Co. (Suzhou, China). The absorbance value A was read at wavelengths of 665, 649, and 470 nm, and the chlorophyll a (Chla), chlorophyll b (Chlb), total chlorophyll (Chl), and carotenoid (Car) contents determined according to Equations (1–5) below:


(1) $$\begin{array}{l} \mathrm{Chla content (mg/g fresh weight) =}\\ \left[\left(13.95\times \Delta A665\right)-\left(6.88\times \Delta A649\right)\right]\times \frac{V\times D}{1,000\times W} \end{array}$$

(2) $$\begin{array}{l} \mathrm{Chlb content (mg/g fresh weight) =}\;\\ {}[(24.96\times \Delta A649-7.32\times \Delta A665]\times \frac{V\times D}{1,000\times W} \end{array}$$

(3) $$\begin{array}{l} \mathrm{Chl content (mg/g fresh weight) =}\\ \left[\left(6.63\times \Delta A665\right)+\left(18.08\times \Delta A649\right)\right]\times \frac{V\times D}{1,000\times W} \end{array}$$

(4) $$\mathrm{Car content (mg fresh weight)}\;=\;C_{x}\times \frac{V\times D}{1,000\times W}$$

(5) $${\mathrm{Car (C}}_{x})\;=\;\frac{\left[(-\left(2.05\times Ca\right)-\left(114.8\times Cb\right)\right]}{245}$$

#### 2.5.3 Determination of plant defense enzyme activities

The collected leaves of American ginseng were immediately transported back to the laboratory with liquid nitrogen preservation and stored in a refrigerator at −80°C before the determination of peroxidase (POD) and ascorbate peroxidase (APX) activities in the leaves. POD and APX activities in the ginseng leaves were measured via visible spectrophotometry using the corresponding enzyme activity kits, including the POD assay (G0107F) and APX activity assay (G0203F) kits (Suzhou Grace Biotechnology Co.).

#### 2.5.4 Disease severity index

Each treatment was performed with three replicates, and the average distribution randomly taken for each replicate of 100 plants (a total of 300 plants). The number of diseased plants were counted for every 10 days of the investigation until the wilting stage, and the incidence rate, disease index, and other indices also observed. The disease grade was divided into six levels, and the specific grading standards used are shown in Table 1. The incidence rate, disease index, and effect of the control were calculated according to Equations (6–8) below, respectively:


(6) $$\begin{array}{l} \mathrm{Incidence of}\;a\;\mathrm{disease}\;\\ \;\left(\%\right)=\frac{\mathrm{Number of incidence plants}}{\mathrm{Number of plants surveyed}}\times 100 \end{array}$$

(7) $$\begin{array}{l} \mathrm{Disease index}=\\ \Sigma \frac{\mathrm{Number of diseased plants}\times \mathrm{Disease level value}}{\mathrm{Total number of plants surveyed}\times \mathrm{Highest disease level value}}\times 100 \end{array}$$

(8) $$\begin{array}{l} \mathrm{Control effect () =}\;\\ \frac{\mathrm{Control of disease index}-\mathrm{Treatment condition index}}{\mathrm{Control of disease index}}\times 100 \end{array}$$

**Table 1:** Classification standards of surface diseases.

Grade	Symptom of a disease
0	Health
1	The proportion of diseased spot area to leaf area was less than or equal to 5%
2	The area of the diseased spot is more than 5% of the leaf area and less than or equal to 10%
3	The area of the disease spot is more than 10% of the leaf area and less than or equal to 20%
4	The area of the disease spot is more than 20% of the leaf area and less than or equal to 50%
5	The diseased spot area is more than 50% of the leaf area

### 2.6 Analysis of the plant microbiome

Fifteen days after the final treatment, *P. quinquefolius* was collected using a five-point sampling method with three replications. The ginseng was then divided into roots and above-ground parts, and all samples surface-sterilized with 75% alcohol for 2 min, washed twice with distilled water, and then quickly frozen in liquid nitrogen, followed by storage at −80°C prior to detecting its microbial diversity. High-throughput Illumina sequencing was used to characterize the microbial community structure in the plant samples (Majorbio Bio-Pharm Technology Co., Shanghai, China). Using primers 799F (5′-AACMGGATTAGTACCCKG-3′) and 1193R (5′-ACGTCATCC CCACCTTCC-3′) (Bulgarelli et al., 2015), the V3–V4 region of the bacterial 16S rRNA gene from American ginseng was amplified. Using primers ITS1F (5′-CTTGGTCATTTAGAGGAAGTAA-3′) and ITS2R (5′-GCT) (GCGTTCTTCATCGATGC-3′) (Sun et al., 2018), the ITS1F–ITS2R region of the fungal gene from American ginseng was amplified. Specific primers with barcodes were synthesized according to the indicated sequencing regions, and the samples then amplified using a thermal cycler (GeneAmp 9700; ABI, USA).

### 2.7 Bioinformatics analysis

16S rRNA and ITS gene sequencing reads were demultiplexed using Fastp (version 0.19.6), merged with FLASH (version 1.2.11), and operational taxonomic units with 97% similarity clustered using UPARSE (version 11). Taxonomic assignments were made using the bacterial SILVA reference (version 138) and fungal UNITE (version 8.0) databases. Alpha diversity was determined using Mothur v.1.30.2. The basic R package “stats” (version 3.3.1.) was used to perform a two-tailed Wilcoxon rank-sum test (wilcox.test function). For high-throughput Illumina sequencing data analysis, the Majorbio Cloud online platform was used.

### 2.8 Data processing and statistical analysis

Data for this trial were organized using Microsoft Excel 2019. SPSS 19.0 was used to test for significant differences in the means (*p* < 0.05) and for correlation analysis. Origin 2021 was used for graphing.

## 3 Results

### 3.1 Morphological indicators

To determine the effects of osthole and *B. amyloliquefaciens* on *P. quinquefolius*, plant height, stem thickness, root dry weight, and root thickness were measured (Table 2). Treatment with CLS and LKWS significantly increased the plant height, stem thickness, root dry weight, and root thickness of *P. quinquefolius* compared to those after treatment with CK. Specifically, CLS significantly (*p* < 0.05) increased the plant height, stem thickness, root dry weight, and root thickness of *P. quinquefolius* by 9.03, 10.63, 61.67, and 25.57%, respectively, and LKWS significantly (*p* < 0.05) increased these by 4.51, 12.94, 62.81, and 22.57%, respectively.

**Table 2:** Effect of Osthole and *Bacillus amyloliquefaciens* on morphological indexes of American ginseng.

Treatment	Plant height (cm)	Stem thick (mm)	Root dry heavy (g)	Root thick (cm)
CK	11.08 ± 0.6596c	0.1715 ± 0.0138b	0.5413 ± 0.0950b	8.37 ± 0.8730b
CLS	12.08 ± 1.1989a	0.1919 ± 0.0152a	0.8751 ± 0.1222a	10.51 ± 0.9899a
LKWS	11.58 ± 0.8651b	0.1937 ± 0.0133a	0.8813 ± 0.0616a	10.29 ± 0.8199a

Data are mean ± standard deviation, different lowercase letters indicate statistically significant data between groups (*P* < 0.05).

### 3.2 Effectiveness of anthrax control

As shown in Table 3, the control treatment (CK) had the highest disease incidence of *P. quinquefolius* anthracnose (16.67%), whereas CLS and LKWS treatment significantly (*p* < 0.05) reduced this, showing lower disease incidence (3.67 and 2.13%, respectively). Consequently, both CLS and LKWS showed good control of western ginseng anthracnose, with CLS showing better control by 76.52%.

**Table 3:** Preventive effect of Osthole and *Bacillus amyloliquefaciens* against the occurrence of American ginseng anthrax.

Treatment	Disease incidence (%)	Disease severity (%)	Control efficacy (%)
CK	16.67 ± 9.02a	9.07 ± 3.21a	–
CLS	3.67 ± 1.53b	2.13 ± 1.62b	76.52
LKWS	9.00 ± 1.00ab	3.67 ± 0.31b	59.54

Data are mean ± standard deviation, different lowercase letters indicate statistically significant data between groups (*P* < 0.05).

### 3.3 Chlorophyll content

**Figure 1:**
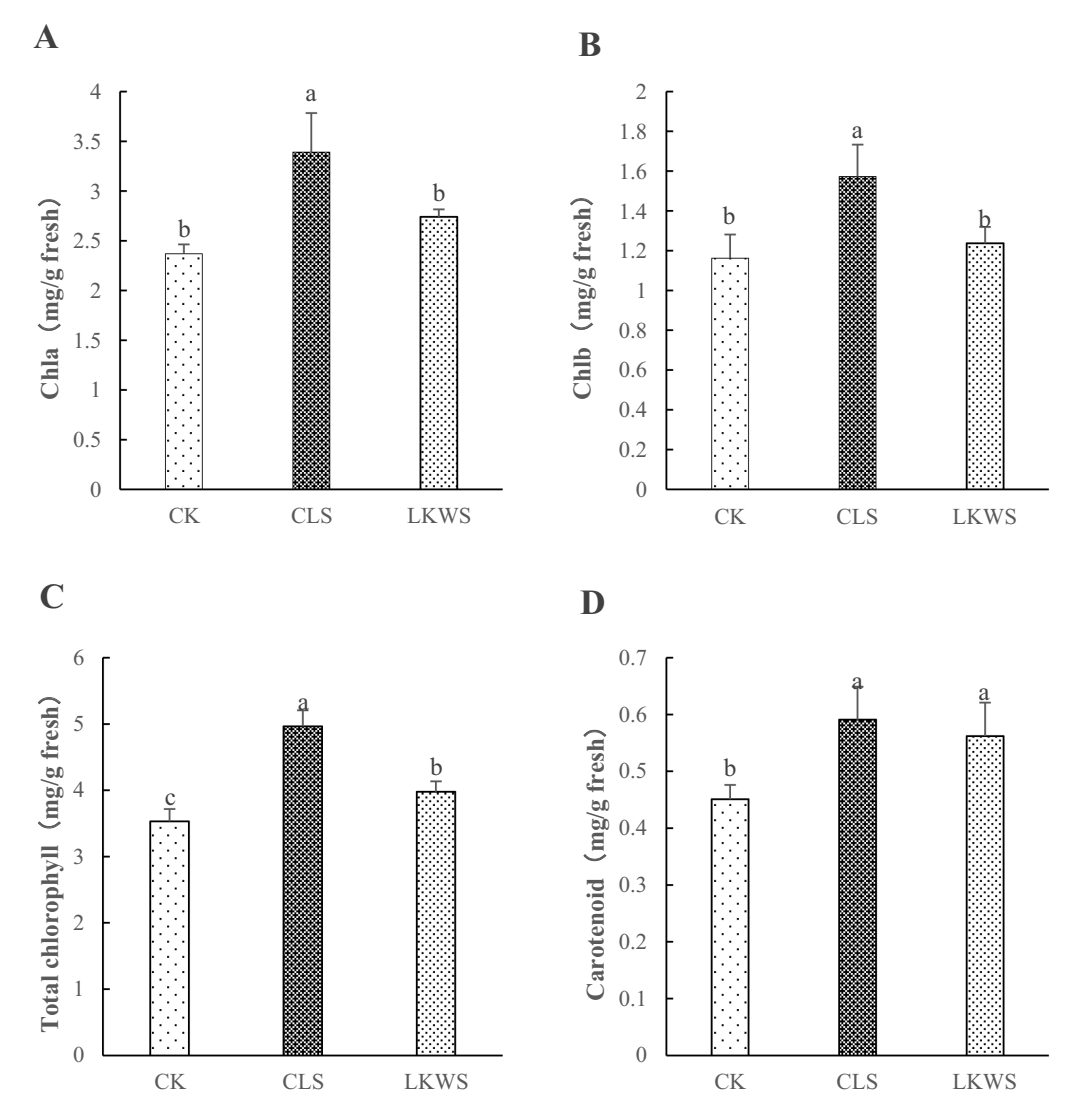
Effect of osthole and *Bacillus amyloliquefaciens* chlorophyll content. Effect of osthole and *Bacillus amyloliquefaciens* on **(A)** Chlorophyll A, **(B)** Chlorophyll B, **(C)** Total chlorophyll and **(D)** Carotenoid. Different lowercase letters indicate significant difference (*p* < 0.05).

The CLS and LKWS treatments had significant effects on the chlorophyll content of *P. quinquefolius* in the forest (Figure 1). CLS significantly (*p* < 0.05) increased the content of Chla and Chlb in *P. quinquefolius* by 43.13 and 35.33%, respectively, compared to that of CK. LKWS did not significantly (*p* < 0.05) increase the content of Chla and Chlb in *P. quinquefolius*; however, they were increased by 15.63 and 6.45%, respectively, compared to that of CK (Figures 1A,B). CLS and LKWS significantly (*p* < 0.05) increased the Chl content of *P. quinquefolius* by 40.57 and 12.61%, respectively, compared to that of CK (Figure 1C). Compared to that of CK, CLS and LKWS significantly (*p* < 0.05) increased the Car content of *P. quinquefolius* by 31.04 and 24.61%, respectively (Figure 1D).

### 3.4 Photosynthesis parameters

Compared to those of CK, treatment with CLS significantly (p < 0.05) increased the P_n_ by 67.96% and significantly decreased the G_s_, C_i_, and T_r_ by 28.57, 9.83, and 18.18%, respectively (Table 4). Compared to those of CK, LKWS significantly (*p* < 0.05) increased the P_n_ and T_r_ by 13.02 and 14.97%, respectively, and significantly (*p* < 0.05) decreased the Gs by 14.29%.

**Table 4:** Effect of Osthole and *Bacillus amyloliquefaciens* on photosynthetic parameters of American ginseng.

Treatment	P_n_ (μmol·m^−2^·s^−1^)	G_s_ (μmol·m^−2^·s^−1^)	C_i_ (μmol·m^−2^·s^−1^)	T_r_ (μmol·m^−2^·s^−1^)
CK	2.84 ± 0.15c	0.14 ± 0.01a	351.46 ± 6.73a	1.87 ± 0.06a
CLS	4.77 ± 0.13a	0.10 ± 0.01c	316.90 ± 10.55b	1.53 ± 0.03c
LKWS	3.21 ± 0.03b	0.12 ± 0.02b	345.67 ± 8.12a	2.15 ± 0.12a

Data are mean ± standard deviation, different lowercase letters indicate statistically significant data between groups (*P* < 0.05).

### 3.5 Antioxidant enzyme activity

**Figure 2:**
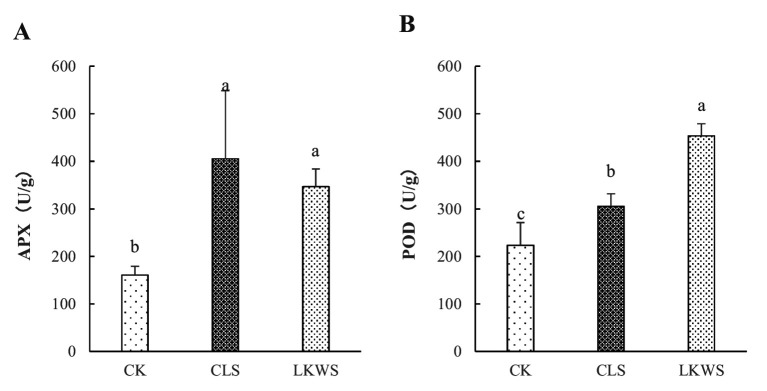
Effects of osthole and *Bacillus amyloliquefaciens* against oxidase activity. Effect of Osthole and Bacillus on **(A)** APX and **(B)** POD enzymes. Different lowercase letters indicate significant difference (*p* < 0.05).

The application of CLS and LKWS significantly (*p* < 0.05) increased the activity of antioxidant enzymes in *P. quinquefolius* (Figure 2). Compared to that of CK, CLS and LKWS significantly (*p* < 0.05) increased the activity of APX enzymes in *P. quinquefolius* by 151.79 and 115.41%, respectively. Moreover, the use of CLS and LKWS significantly (*p* < 0.05) increased POD activity in *P. quinquefolius* by 36.78 and 102.93%, respectively, compared to that of CK.

### 3.6 Microbiome analysis of aboveground parts and the roots of *Panax quinquefolius*

**Figure 3:**
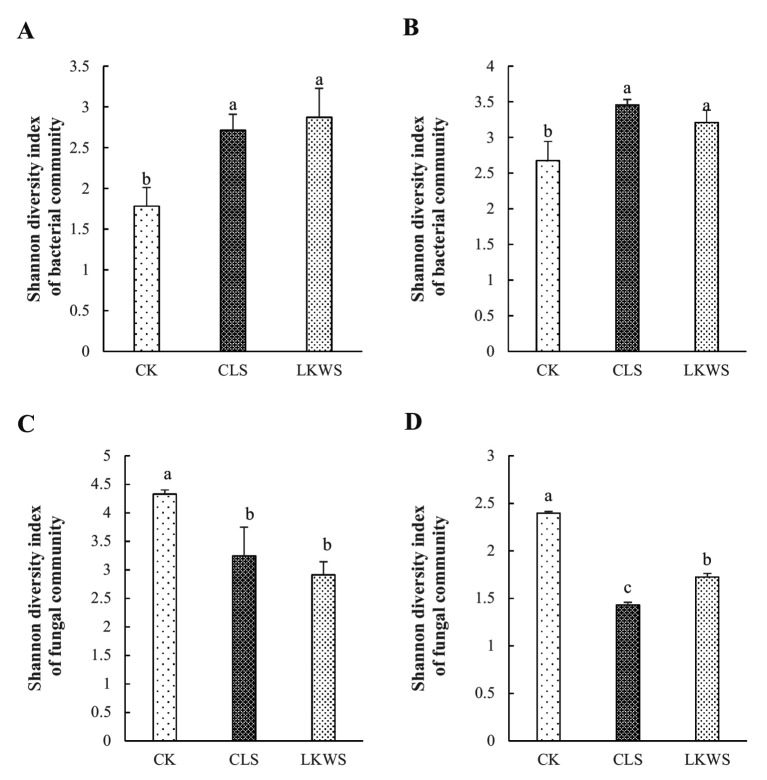
Shannon diversity index of the bacterial community structure of the **(A)** aboveground and **(B)** root of American ginseng and Shannon diversity index of the fungal community structure of the **(C)** aboveground and **(D)** root of American ginseng. Different lowercase letters indicate significant difference (*p* < 0.05).

Shannon’s index was used to assess microbial diversity of the aboveground and root parts of *P. quinquefolius* under the different treatment conditions (Figure 3). Compared with that of CK, CLS increased the bacterial diversity in aboveground and root parts of *P. quinquefolius* by 52.29 and 29.11%, respectively, and LKWS increased these by 61.25 and 19.92%, respectively. Compared with that of CK, CLS significantly decreased fungal diversity in the aboveground and root parts of *P. quinquefolius* by 25.07 and 40.34%, respectively, and LKWS significantly decreased these by 32.70 and 28.04%, respectively.

**Figure 4:**
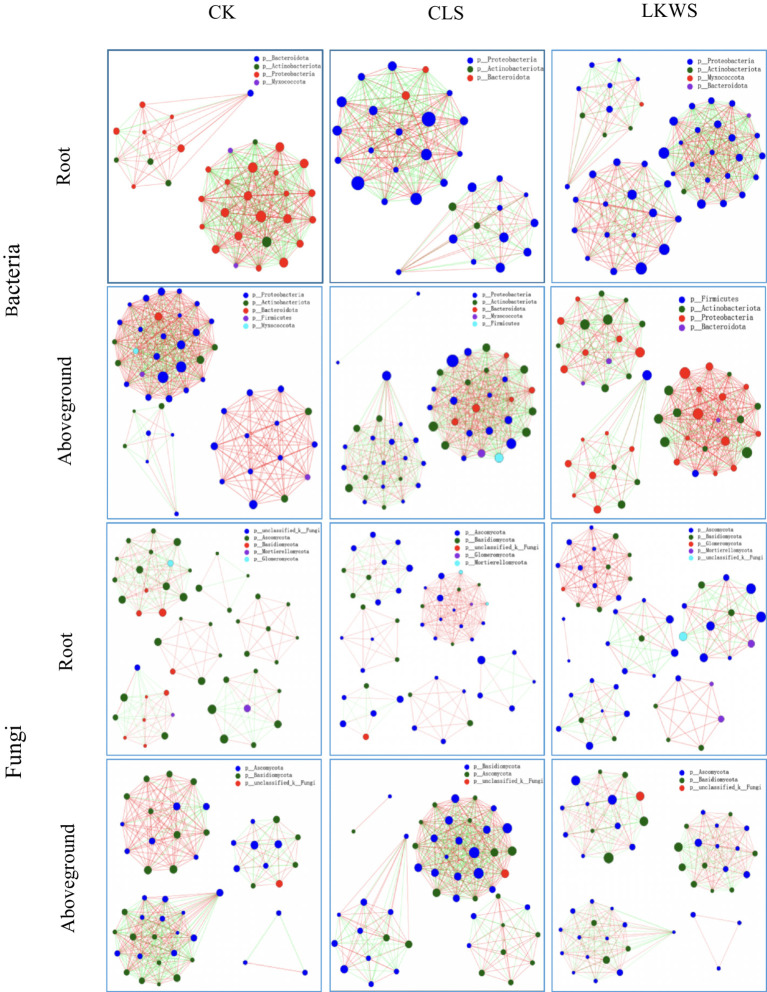
Network analysis under different treatments of American ginseng. The size of the nodes in the figure indicates the abundance of the species, different colors indicate different species; the color of the line indicates positive and negative correlation, red indicates positive correlation, green indicates negative correlation; the thickness of the line indicates the size of the correlation coefficient, the thicker the line, the higher the correlation between species; the more lines, the closer the connection between the species and other species.

Symbiotic networks of bacterial and fungal communities varied among the different parts of *P. quinquefolius* and among treatments (Figure 4, Tables 5, 6). With the exception of the CLS treatment, the structure of the bacterial network in the roots of *P. quinquefolius* was typically more complex than that in the aboveground parts, based on the number of edges and nodes, as well as the average extent. Among all the bacterial networks, that of the CLS treatment was the simplest in the roots of *P. quinquefolius* (nodes: 352; edges: 10721: average degree: 60.915). In the aboveground parts and roots of *P. quinquefolius*, both CLS and LKWS treatments had higher positive and lower negative correlations than those of CK.

**Table 5:** Key topological characteristics of bacterial networks in ground and roots of American ginseng.

	Aboveground			Root		
	CK	CLS	LKWS	CK	CLS	LKWS
Nodes	431	490	392	437	352	461
Edges	15,826	23,242	14,778	16,051	10,721	19,010
Positive edges ratio (%)	67.01	79.56	84.4	66.82	79	78.04
Negative edges ratio (%)	22.99	20.44	15.6	33.18	21	21.96
Average degree	73.439	94.865	75.398	73.460	60.915	82.473

**Table 6:** Key topological characteristics of fungal networks in ground and roots of American ginseng.

	Aboveground			Root		
	CK	CLS	LKWS	CK	CLS	LKWS
Nodes	500	499	500	147	85	92
Edges	25,204	22,046	24,386	1993	934	736
Positive edges ratio (%)	59.90	70.82	80.73	90.22	95.5	89.95
Negative edges ratio (%)	40.10	29.18	19.27	9.78	4.5	10.05
Average degree	100.816	88.361	97.544	27.116	21.976	16

The structure of fungal networks in the aboveground parts of *P. quinquefolius* was generally more complex than that in the roots, based on the number of edges and nodes, as well as the average degree when compared to the bacterial networks (Figure 4, Tables 5, 6). Nodes, edges, and the average extent of fungal networks were lower in the aboveground parts and roots in the CLS treatment than in the CK. Edges and the average extent of fungal networks were lower in aboveground parts and roots of *P. quinquefolius* in LKWS treatments compared to those of CK, but no differences were observed in the nodes of the aboveground parts.

**Figure 5:**
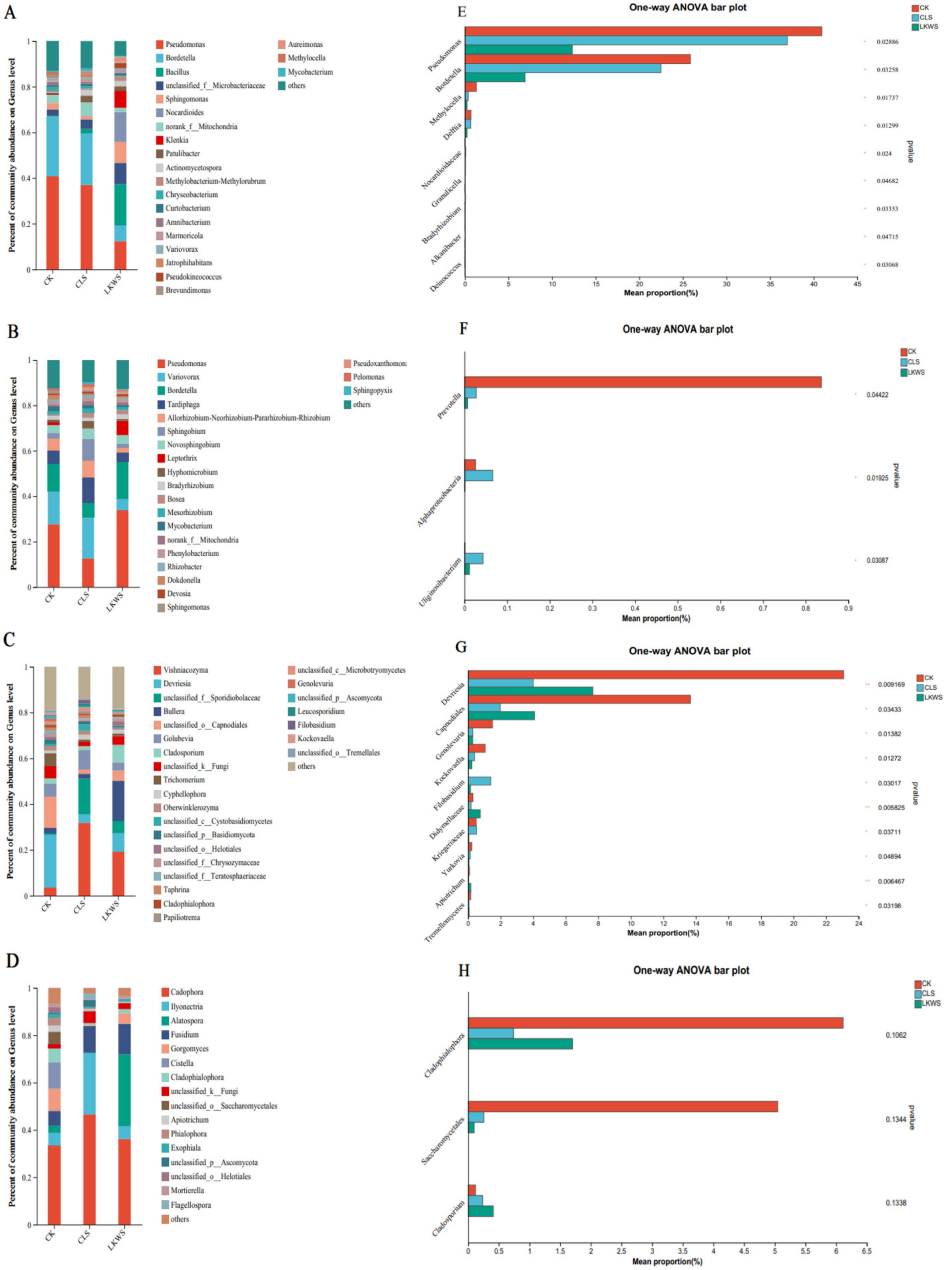
Microbial composition and difference analysis of aerial parts and roots of American ginseng. Relative abundance at the bacterial level of aerial parts **(A)** and root **(B)**; relative abundance of aerial parts **(C)** and root **(D)**. The abscissa is the treatment, the ordinate is the proportion of the genus in the sample, the columns of different colors represent different species, and the length of the pillars represents the size of the proportion of the species. One-way analysis of variance (ANOVA) was used to test differences between the bacterial levels of the ground **(E)** and root **(F)** and the ground **(G)** and root **(H)**. The y-axis represents species names at the genus level, the x-axis represents the mean relative abundance of different species groups, and columns of different colors represent different groups. On the far right is the value of *p*, * *p* < 0.05 * * *p* < 0.01 * * * *p* < 0.001.

Microorganisms of the aboveground parts and roots of *P. quinquefolius* were observed and constructed using bacterial and fungal genera with relative abundances greater than 1%, respectively, to assess the relationship between microorganisms in different parts of *P. quinquefolius* and under different treatments (Figure 5). The relationship between bacteria and different treatments in the aboveground parts of *P. quinquefolius* is shown in Figure 5A. *Pseudomonas* (40.94%) and *Bordetella* (25.87%) were the main bacterial genera present in the CK treatment in the aboveground parts of *P. quinquefolius*. Similarly, the main bacterial genera in the aboveground parts of *P. quinquefolius* in the CLS treatment were *Pseudomonas* (36.99%) and *Bordetella* (22.48%), whereas those in the LKWS treatment were *Bacillus* (18.11%), *Nocardioides* (12.97%), and *Pseudomonas* (12.31%). The relationship between the bacteria in the roots of ginseng and the different treatments is shown in Figure 5B. *Pseudomonas* (27.65%), Var*iovorax* (14.44%), and *Bordetella* (12.30%) were the dominant bacterial genera in the CK treatment in the roots of *P. quinquefolius*. The dominant bacterial genera in the CLS treatment in the roots of *P. quinquefolius* were *Variovorax* (17.91%), *Pseudomonas* (12.56%) and *Tardiphaga* (11.20%), whereas those in the LKWS treatment were *Pseudomonas* (33.79%) and *Bordetella* (16.52%). The relationship between fungi in the aboveground parts of *P. quinquefolius* and the different treatments is shown in Figure 5C. The main fungal genera present in aboveground parts of *P. quinquefolius* in the CK treatment were *Devriesia* (23.12%) and *Capnodiales* (13.65%). The main fungal genera in aboveground parts of *P. quinquefolius* in the CLS treatment were *Vishniacozyma* (31.82%) and *Sporidiobolaceae* (15.53%), whereas those of the LKWS treatment were *Vishniacozyma* (19.23%) and *Bullera* (17.64%). The relationship between fungi in the roots of *P. quinquefolius* and the different treatments is shown in Figure 5D. The main fungal genera in the roots of *P. quinquefolius* in the CK treatment were *Cadophora* (33.42%) and *Cistella* (11.21%). The main fungal genera in the roots of *P. quinquefolius* in the CLS treatment were *Cadophora* (46.57%), *Ilyonectria* (25.85%), and *Fusidium* (11.18%), whereas those of the LKWS were *Cadophora* (36.18%), *Alatospora* (30.44%), and *Fusidium* (12.96%).

Microorganisms in American ginseng differed significantly among the different treatments. In the aboveground parts of *P. quinquefolius*, the bacterial genera, *Pseudomonas* and *Bordetella*, were significantly higher in the CK treatment than in the CLS and LKWS treatments (Figure 5E). In the roots of American ginseng, the bacterial genus, *Prevotella*, was significantly higher in the CK treatment than in the CLS and LKWS treatments, and the bacterial genera, *Alphaproteobacteria* and *Uliginosibacterium*, significantly more abundant in the CLS treatment than in the other treatments (Figure 5F). In the aboveground portion of *C. occidentalis*, the fungal genera, *Devriesia* and *Capnodiales*, were significantly more abundant in the CK treatment than in the other treatments (Figure 5G).

## 4 Discussion

The present study showed that the use of osthole and *B. amyloliquefaciens* significantly (*p* < 0.05) improved the growth of *P. quinquefolius* in a forest (Table 2), where their application significantly increased the plant height, stem thickness, root dry weight, and root thickness. The improvement of these growth indicators not only reflects the growth status of the plant, but also closely related to the physiological and ecological adaptations of the plant, which can directly affect the growth, development and yield of the plant. Studies have shown that plant height and stem thickness are important reflections of the competitive ability of plants, and higher plant height helps to obtain more light, thus promoting photosynthesis (Rebitanim et al., 2020). Root dry weight and root thickness are related to the water and nutrient uptake capacity of plants, and the enhancement of root development can improve the adaptability of plants to adverse environments (Comas et al., 2013). Our results are in line with those of previous studies on the promotion of coumarin production in faba beans (Saleh et al., 2015) and the growth promotion of *Arabidopsis thaliana* by *B. amyloliquefaciens* (Lu et al., 2021). The growth-promoting effect of osthole, a derivative of coumarin, on *Codonopsis* may affect plant growth by interfering with phytohormone metabolism (Cheynier et al., 2013). Plant growth can be affected by *B. amyloliquefaciens* via the secretion of IAA (Ji et al., 2021), which promotes nitrogen fixation by plant roots (Abdallah et al., 2018), solubilizes phosphates (Vinci et al., 2018) and (Jiang et al., 2015), generates iron carriers (Dimopoulou et al., 2021), among others, to promote plant growth. Together, these mechanisms of action promoted the growth of *Panax quinquefolius*, further validating the importance of growth indicators in assessing plant growth status and production potential.

Chlorophylls are the most abundant pigments in the photosynthetic system of land plants and algae and are indispensable for the absorption of light energy and transfer of electrons in photosynthesis (Hu et al., 2021). Carotenoids are necessary for leaf photosynthesis and photoprotection (Rodriguez-Concepcion and Daròs, 2022). In our study, the use of osthole and *Bacillus amyloliquefaciens* significantly increased chlorophyll and carotenoid content (Figure 1). Photosynthesis plays a crucial role in the synthesis and accumulation of organic matter, plant growth, nutrient uptake, and responses to abiotic and biotic stress (Bunce, 2008). Our results showed that the use of osthole significantly increased the P_n_ (Table 4), which may be due to the fact that osthole significantly increased the chlorophyll content of American ginseng. Compared with those of CK, the use of *B. amyloliquefaciens* also significantly increased the P_n_ and T_r_, which is similar to the results of a previous study on alfalfa seedlings (Han et al., 2022). These results indicate the promising application of osthole and *Bacillus amyloliquefaciens* and its use in the optimization of photosynthesis efficiency. In the present study, the use of both osthole and *B. amyloliquefaciens* significantly reduced the G_s_, which may be due to the ability of the plant to avoid excessive water loss by closing stomata (Novick et al., 2016). This further illustrates the potential of osthole and *Bacillus amyloliquefaciens* in water regulation.

Numerous studies have shown that antioxidant enzymes play a great role in alleviating the accumulation of reactive oxygen species (ROS) and reducing oxidative stress in plants (Han et al., 2022). APX and POD are the main antioxidant enzymes that convert H_2_O_2_ to H_2_O (Ren Y. et al., 2020; Rosa et al., 2010). The present study showed that the use of osthole and *B. amyloliquefaciens* significantly increased the activities of APX and POD in *P. quinquefolius*. This result suggests that they may be effective in enhancing the antioxidant defense system of *Panax quinquefolius*, thereby improving its tolerance to oxidative stress. Coumarin has the function of enhancing the antioxidant defense system (Wu et al., 2009), and osthole, a naturally occurring coumarin, has the potential to inhibit the production of active oxidants (Tsai et al., 2015), which may explain its ability to enhance the antioxidant enzyme activity of forest ginseng. The use of *B. amyloliquefaciens* has previously been shown to enhance APX and POD activities in tomatoes (Wang et al., 2019), which is consistent with our results.

In addition, we investigated the effects of osthole and *B. amyloliquefaciens* on the *P. quinquefolius* microbiome. The structure of the plant microbiome is influenced by complex interactions among the host, microorganisms, and other relevant environmental factors (Dastogeer et al., 2020). Our results showed that the use of osthole and *B. amyloliquefaciens* significantly increased the diversity of bacteria in the aboveground parts and roots of *P. quinquefolius*, while significantly decreasing the diversity of fungi (Figure 3). The significant decrease in fungal diversity of *P. quinquefolius* may be due to its strong antifungal activity (Guo et al., 2021). Previously, the use of *B. amyloliquefaciens* was shown to significantly increase ginseng bacterial diversity and decrease its fungal diversity (Tian et al., 2018), which is consistent with the results of our study. Interactions with microorganisms can affect community stability (Coyte et al., 2015). Our results showed that CLS was more complex than CK was in the bacterial network of the aboveground parts of sago ginseng; LKWS was more complex than CK was in the bacterial network of sago ginseng roots; and CK was more complex than CLS and LKWS were in the fungal network based on edges and nodes (Figure 4, Tables 5, 6). This suggests that the use of osthole and *B. amyloliquefaciens* can increase the complexity of bacterial communities while decreasing the complexity and improving the stability of fungal communities. The complexity of microbial networks may be related to the alpha diversity (Fan et al., 2018). Previous studies have shown that *Pseudomonas* and *Bordetella* are potential pathogens (Hamidou Soumana et al., 2017; Fernández et al., 2015), where our study showed that the use of CLS and LKWS significantly reduced their abundance when compared to that of CK (Figure 5E), which may also explain the reduction observed in disease incidence and severity in *C. occidentalis* after the use of osthole and deconjugated *B. amyloliquefaciens*. Previous studies have identified Var*iovorax* as a class of plant growth-promoting inter-root bacteria (Han et al., 2011) that enhances host plant resilience and disease resistance (Belimov et al., 2009; Belimov et al., 2005). *Tardiphaga* plays an important role in the N cycle (Alves et al., 2014). Our results suggest that *Variovorax* and *Tardiphaga* are the main bacterial genera of CLS in the roots of *P. quinquefolius* (Figure 5B), which may explain why the use of osthole promotes the growth of *P. quinquefolius* while enhancing stress and disease resistance. Previous studies have shown the potential growth-promoting effects of *Alphaproteobacteria* (Pini et al., 2011), and that *Uliginosibacterium* contributes to biofilm formation (Jiao et al., 2021). The bacterial genera, *Alphaproteobacteria* and *Uliginosibacterium*, were significantly higher in CLS than in other treatments tested in our study (Figure 5F). Some potential pathogenic bacteria, such as *Devriesia* and *Capnodiales*, were significantly lower than those in CK after both CLS and LKWS treatments (Abdollahzadeh et al., 2020; Li et al., 2013) (Figure 5G). The relative abundance of some potential plant growth-promoting microorganisms, such as *Vishniacozyma*, *Cadophora*, and *Alatospora*, was higher than that of CK in the CLS and LKWS treatments (Figures 5C,D) (Artigas et al., 2017; Bizabani and Dames, 2015; Lutz et al., 2020). Therefore, we hypothesized that the application of osthole and *B. amyloliquefaciens* may recruit plant growth-promoting microorganisms by stimulating *C. occidentalis* and providing more nutrients for plant growth while inhibiting the invasion and proliferation of potential pathogens (Wang et al., 2022).

## 5 Conclusion

In this study, the application of osthole and *B. amyloliquefaciens* to understory ginseng revealed that their use improved the growth of understory American ginseng by enhancing photosynthetic capacity, stimulating the activity of antioxidant enzymes to increase the tolerance of *P. quinquefolius*, and promoting the accumulation of plant biomass. In addition, the use of osthole and *B. amyloliquefaciens* altered the structure of the microbial community of *P. quinquefolius*, significantly increased the diversity of *P. quinquefolius* bacteria, significantly decreased the diversity of *P. quinquefolius* fungi, and stimulated the recruitment of more growth-promoting microorganisms into the American ginseng to build a more stable microbial network, which resulted in a significant decrease in the incidence of *P. quinquefolius* anthracnose and the index of the disease. Therefore, based on the above results, it was shown that the use of osthole is an effective way to improve the growth of *P. quinquefolius* in the forest and, at the same time, provides a theoretical basis for its effective application in agriculture. In order to further deepen the research and promote the agricultural dissemination of osthole, future work could focus on (1) analyzing in depth the specific mechanisms by which osthole affects the microbial community of American ginseng and further verifying the relationship between these community changes and disease resistance; (2) evaluating the potential for the application of osthole in other cash crops, especially in terms of reduction of chemical pesticides and enhancement of crop resistance to disease, in order to explore its broader applicability.

## Data Availability

The data presented in the study are deposited in the NCBI repository, accession number PRJNA1189344.
